# Etesevimab in combination with JS026 neutralizing SARS-CoV-2 and its variants

**DOI:** 10.1080/22221751.2022.2032374

**Published:** 2022-02-10

**Authors:** Fengze Wang, Li Li, Yang Dou, Rui Shi, Xiaomin Duan, Hongchuan Liu, Jing Zhang, DanDan Liu, Jing Wu, Yang He, Jun Lan, Bai Lu, Hui Feng, Jinghua Yan

**Affiliations:** a CAS Key Laboratory of Pathogenic Microbiology and Immunology, Institute of Microbiology, Chinese Academy of Sciences, Beijing, People’s Republic of China; b University of Chinese Academy of Sciences, Beijing, People’s Republic of China; c Shanghai Junshi Biosciences Co. Ltd, Shanghai, People’s Republic of China; d School of Pharmaceutical Sciences, IDG/McGovern Institute for Brain Research, Tsinghua University, Beijing, People’s Republic of China; e Beijing Advanced Innovation Center for Structural Biology, School of Life Sciences, Tsinghua University, Beijing, People’s Republic of China

**Keywords:** SARS-CoV-2, etesevimab, JS026, antibody cocktail, variants of concern

## Abstract

The neutralizing antibody is a potential therapeutic for the ongoing COVID-19 pandemic. As an antiviral agent, numerous mAbs recognize the epitopes that overlap with ACE2-binding sites in the SARS-CoV-2-RBD. Some studies have shown that residual changes on the spike protein can significantly decrease the efficiency of neutralizing antibodies. To address this issue, a therapeutic cocktail could be an effective countermeasure. In the present study, we isolated a fully human neutralizing antibody, JS026, from a convalescent patient. The comparative analysis revealed that JS026 binding to SARS-CoV-2-RBD mainly located between epitopes for class 2 and class 3 mAbs as opposed to that of class 1 (etesevimab) antibodies. A cocktail of etesevimab and JS026 increased neutralizing efficacy against both wild-type SARS-CoV-2 and the recent emergence of Alpha, Beta, Gamma, and Delta variants. JS026 and the cocktail reduced virus titers in the infected lungs of hACE2 transgenic mice and relieved pathological changes. These findings would benefit antibody-based therapeutic countermeasures in the treatment of COVID-19.

## Main text

The power of monoclonal antibody (mAb) treatment for the deadly virus has been widely acknowledged [1,2]. Since the initial outbreak of coronavirus disease 2019 (COVID-19) in late 2019, mAbs-based approaches hold enormous promise for the treatment of COVID-19. The phase 3 BLAZE-1 clinical trial shows that early administration bamlanivimab and etesevimab (also named CB6, JS016, or LY-CoV016) accelerated the decline in the SARS-CoV-2 viral load and led to a lower incidence of COVID-19-related hospitalization by 70% and mortality by 100% [3]. Emergency use authorizations (EUA) were authorized for bamlanivimab and etesevimab administered together to treat individuals of mild to moderate symptoms at high risk for progression to severe COVID-19 and post-exposure prophylaxis of COVID-19 [4]. In recent months, the neutralizing activity of bamlanivimab has been markedly diminished due to the emergence of SARS-CoV-2 variants of concern (VOCs), beta, gamma, and delta strains, which has become the predominant strain in this pandemic [5]. Identifying a new neutralizing antibody (NAb) that could replace bamlanivimab to combat these VOCs has become an urgent matter. It has been shown that NAbs that do not compete for binding to the RBD exhibit exceptionally potent neutralization activities to SARS-CoV-2 and VOCs [6]. Discovering such a new NAb to combine with etesevimab may be a good strategy to develop broadly effective therapies to limit morbidity and mortality of the devastating COVID-19 VOCs currently ongoing.

**Figure 1.**
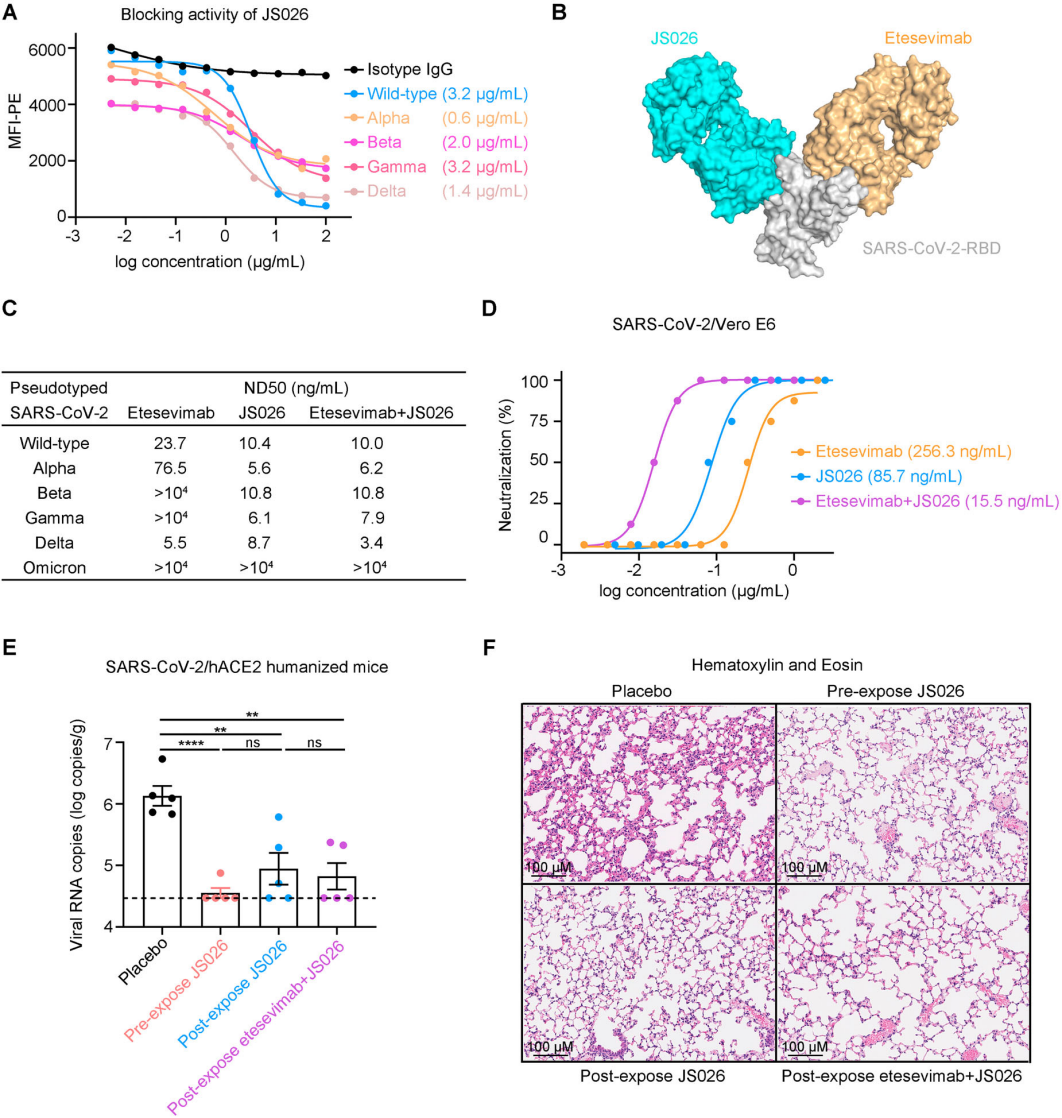
Synergistic effects of JS026 and etesevimab in neutralizing SARS-CoV-2 VOCs. (a) mAb JS026 potently inhibited the binding of RBD from WT SARS-CoV-2 or VOCs to hACE2. HEK293T-hACE2 cells were stained with SARS-CoV-2-RBD proteins pre-incubated with isotype IgG or JS026 in flow cytometry-based assay. (b) Superimposition of JS026/RBD complex and etesevimab/RBD (PDB: 7C01) reveal the non-overlapping epitopes of etesevimab and JS026. The JS026-Fab, etesevimab-Fab, and SARS-CoV-2-RBD are coloured differently as indicated. (c,d) The neutralization activity of mAbs against SARS-CoV-2 VOCs. The mixtures of pseudotyped or authentic SARS-CoV-2 were incubated with serially diluted etesevimab, JS026, or the JS026/etesevimab cocktail. The mixtures were then added to HEK293T-hACE2 or Vero E6 cells for another incubation. One of three independent experiment data is shown. (e) Before SARS-CoV-2 challenge, the hACE2 transgenic mice (*n* = 8) in the prophylactic setting were intravenously infused with JS026. Then, 5 × 10^5^ TCID50 SARS-CoV-2 was intranasally administered to animals and the therapeutic setting was injected with JS026, mAbs cocktail, or PBS as a vehicle control one day after challenging. SARS-CoV-2 titres in the lungs from five mice were measured by qRT-PCR (unpaired *t*-test, ns *p* > 0.05, ** *p* < 0.01, and **** *p* < 0.0001). (f) Histopathology examination of lung tissues (three mice) was performed at 5-day post-infection point. Scale bar: 100 μm.

In this study, we sorted SARS-CoV-2-RBD-specific memory B cells from peripheral blood mononuclear cells (PBMCs) of COVID-19 convalescents and amplified the variable region coding sequences of IgG antibodies as previously reported method [7]. To investigate the blocking breadth and potency of isolated human mAbs, soluble RBDs of currently circulating SARS-CoV-2 VOCs and HEK293T-hACE2 cells were used to perform flow cytometry (FACS) based assays. Strikingly, a potent mAb, JS026, showed broadly blocking all five RBDs binding to ACE2 receptor with a median half-maximal inhibitory concentration (IC50) value of 0.6-3.2 μg/mL (Figure 1(a)). Bio-Layer Interferometry (BLI) assays demonstrated that JS026 bound to the wild-type (WT) RBD protein with a KD of 2.04 nM, to VOCs RBD antigens with similar affinities (Supplementary Table 1). The majority of SARS-CoV-2-neutralizing mAbs authorized or in development are clustered to class 1–4 as structural comparisons revealed these molecules flexibly direct to different epitopes on the RBD [8]. The competition-binding assay indicated JS026 locates between epitopes for class 2 (P2B-2F6) and class 3 (S309) mAbs as opposed to that of class 1 (etesevimab) antibodies (Supplementary Figure 1). To gain insights into the structural basis of the blocking, we solved the crystal structures of JS026-Fab/RBD at a resolution of 2.5 Å (Supplementary Table 2). Structural analyses revealed that JS026 blocks SARS-CoV-2-RBD binding to hACE2 mainly through the steric clash and the mAb inclines to share overlapping sites of class 3 antibodies instead of etesevimab (Figure 1(b) and Supplementary Figure 2). According to the pseudovirus neutralization assay, JS026 exhibited very low 50% neutralization dose (ND50) values of 5.6-10.8 ng/mL for main circulating SARS-CoV-2 VOCs. Importantly, a cocktail of etesevimab and JS026 exhibit exceptionally potent neutralization activities against both WT and SARS-CoV-2 VOCs pseudovirus including the recent emergence of delta variant characterized by increased transmission fitness and decreased sensitivity to preventive measures [9] (Figure 1(c)). Omicron harbours 15 mutations in the RBD including almost all the sites of the existing VOCs. To investigate the breadth of etesevimab, JS026, and cocktail neutralizing activity to pseudotyped Omicron, neutralization assays were conducted in vitro. For etesevimab, the K417N mutation fully decreased its binding ability to the RBD [10]. The structure revealed that N440 forms 29 contacts with JS026 and two hydrogen bonds with A111 (HCDR3) and W32 (LCDR1) of JS026, and its mutation to Lys may evaded the antibody (Supplementary Figure 3). The ND50 of the single NAb or combination is higher than 10 μg/mL (Supplementary Figure 4). Individual NAb, etesevimab or JS026, exhibited ND50s of 256 or 86 ng/mL to authentic SARS-CoV-2 virus, respectively. Interestingly, a combination of etesevimab and JS026 showed greatly enhanced neutralizing potency (ND50 = 15.5 ng/mL), against SARS-CoV-2 (Figure 1(d)). These results indicate etesevimab and JS026 targeting-non-overlapping sites on the RBD exhibited synergistic neutralization *in vitro*.

Next, we tested the efficacy of JS026 and the JS026 and etesevimab cocktail in hACE2 mice challenge models for SARS-CoV-2. In the prophylactic group, animals were administered a single shot of JS026 one day before SARS-CoV-2 infection. The viral RNAs in the lungs were significantly lower than controls and 4 out of 5 (4/5) mice were undetectable 5-days after the infection with SARS-CoV-2 (Figure 1(e)). Receiving JS026 or cocktail at one-day post-intranasal SARS-CoV-2 inoculation, 2/5 mice in single antibody-treated group and 3/5 mice dosed with the antibody cocktail were under the limit of detection at the 5-day post-infection point (Figure 1(e)). In addition to viral RNA copies reduction, we also assessed lung tissue of mice by histochemical staining assays. Control mice showed evidence of mild interstitial pneumonia, which was characterized by the lung parenchyma significantly widened and inflammatory cells infiltrated in subcapsular multifocal alveolar septa around blood vessels (Figure 1(f)). In contrast, animals treated with the antibody cocktail showed minimal evidence of interstitial pneumonia with visible leukocyte infiltrations and slight alveolar septal thickening (Figure 1(f)).

Our studies provided evidence for a cocktail NAbs (etesevimab and JS026) targeting non-overlapping epitopes increase therapeutic efficacy for SARS-CoV-2 VOCs that harbour multiple mutations within the RBD. In November 2021, China National Medical Products Administration (NMPA) has approved the application for a phase I clinical trial with JS026. Etesevimab in combination with JS026 provides a potential treatment option to treat the SARS-CoV-2 VOCs and combat the COVID-19 pandemic.

## Supplementary Material

Supplemental Material

## Data Availability

Further information and requests for resources should be directed to and will be fulfilled by the corresponding authors.

## Code availability statement

The accession number for the atomic coordinates and diffraction data reported in this study is PDB 7F7E.
