# Supplementary material for: Etesevimab in combination with JS026 neutralizing SARS-CoV-2 and its variants
Source: Emerg Microbes Infect. 2022 Feb 10;11(1):548–51. doi: 10.1080/22221751.2022.2032374 (PMC8843163; doi:10.1080/22221751.2022.2032374)
Supplement: Supplemental Material [file TEMI_A_2032374_SM2670.zip › Suppl files/Report.pdf]

Preliminary Full wwPDB X-ray Structure Validation Report (i)

Jun 26, 2021 – 11:15 AM JST

Deposition ID : D_1300022796

This is a Preliminary Full wwPDB X-ray Structure Validation Report.

This report is produced by the wwPDB Deposition System during initial deposition but before annotation of the structure.

We welcome your comments at *validation@mail.wwpdb.org*
A user guide is available at
https://www.wwpdb.org/validation/2017/XrayValidationReportHelp
with specific help available everywhere you see the (i) symbol.

PRELIMINARY VALIDATION REPORT

The following versions of software and data (see references (i)) were used in the production of this report:

MolProbity	:	4.02b-467
Mogul	:	1.8.5 (274361), CSD as541be (2020)
Xtriage (Phenix)	:	1.13
EDS	:	2.20
Percentile statistics	:	20191225.v01 (using entries in the PDB archive December 25th 2019)
Refmac	:	5.8.0158
CCP4	:	7.0.044 (Gargrove)
Ideal geometry (proteins)	:	Engh & Huber (2001)
Ideal geometry (DNA, RNA)	:	Parkinson et al. (1996)
Validation Pipeline (wwPDB-VP)	:	2.20

1 Overall quality at a glance (i)

The following experimental techniques were used to determine the structure:
X-RAY DIFFRACTION

The reported resolution of this entry is 2.49 Å.

Percentile scores (ranging between 0-100) for global validation metrics of the entry are shown in the following graphic. The table shows the number of entries on which the scores are based.

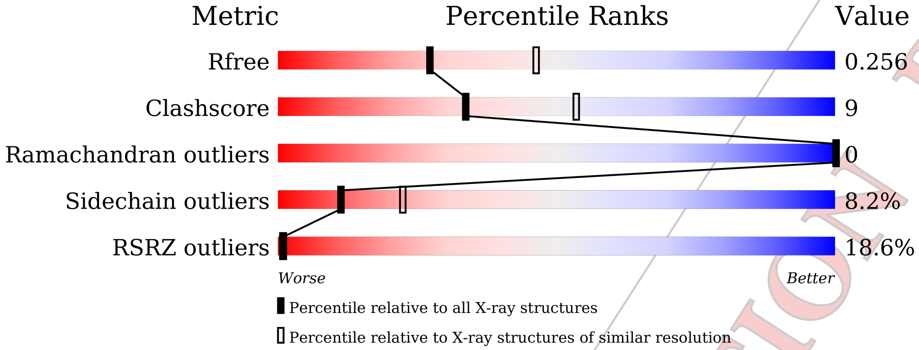

Metric	Whole archive (#Entries)	Similar resolution (#Entries, resolution range(Å))
R_{free}	130704	4661 (2.50-2.50)
Clashscore	141614	5346 (2.50-2.50)
Ramachandran outliers	138981	5231 (2.50-2.50)
Sidechain outliers	138945	5233 (2.50-2.50)
RSRZ outliers	127900	4559 (2.50-2.50)

The table below summarises the geometric issues observed across the polymeric chains and their fit to the electron density. The red, orange, yellow and green segments of the lower bar indicate the fraction of residues that contain outliers for >=3, 2, 1 and 0 types of geometric quality criteria respectively. A grey segment represents the fraction of residues that are not modelled. The numeric value for each fraction is indicated below the corresponding segment, with a dot representing fractions <=5% The upper red bar (where present) indicates the fraction of residues that have poor fit to the electron density. The numeric value is given above the bar.

Mol	Chain	Length	Quality of chain
1	C	227	19% / 77% 22% •
2	L	205	23% / 74% 24% •
3	E	198	13% / 71% 25% • •

PRELIMINARY VALIDATION REPORT

2 Entry composition (i)

There are 4 unique types of molecules in this entry. The entry contains 4835 atoms, of which 0 are hydrogens and 0 are deuteriums.

In the tables below, the ZeroOcc column contains the number of atoms modelled with zero occupancy, the AltConf column contains the number of residues with at least one atom in alternate conformation and the Trace column contains the number of residues modelled with at most 2 atoms.

- Molecule 1 is a protein.

Mol	Chain	Residues	Atoms					ZeroOcc	AltConf	Trace
			Total	C	N	O	S			
1	C	227	1691	1075	277	333	6	0	0	0

- Molecule 2 is a protein.

Mol	Chain	Residues	Atoms					ZeroOcc	AltConf	Trace
			Total	C	N	O	S			
2	L	205	1560	978	264	313	5	0	0	0

- Molecule 3 is a protein.

Mol	Chain	Residues	Atoms					ZeroOcc	AltConf	Trace
			Total	C	N	O	S			
3	E	198	1570	1006	264	292	8	0	0	0

- Molecule 4 is 2-acetamido-2-deoxy-beta-D-glucopyranose (three-letter code: NAG) (formula: $C_8H_{15}NO_6$).

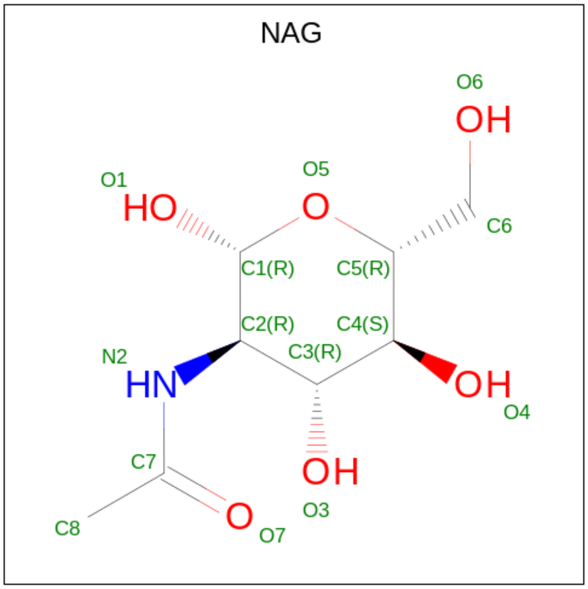

PRELIMINARY VALIDATION REPORT

Mol	Chain	Residues	Atoms				ZeroOcc	AltConf
			Total	C	N	O		
4	E	1	14	8	1	5	0	0

PRELIMINARY VALIDATION REPORT

3 Residue-property plots (i)

These plots are drawn for all protein, RNA, DNA and oligosaccharide chains in the entry. The first graphic for a chain summarises the proportions of the various outlier classes displayed in the second graphic. The second graphic shows the sequence view annotated by issues in geometry and electron density. Residues are color-coded according to the number of geometric quality criteria for which they contain at least one outlier: green = 0, yellow = 1, orange = 2 and red = 3 or more. A red dot above a residue indicates a poor fit to the electron density (RSRZ > 2). Stretches of 2 or more consecutive residues without any outlier are shown as a green connector. Residues present in the sample, but not in the model, are shown in grey.

- Molecule 1:

Chain C:

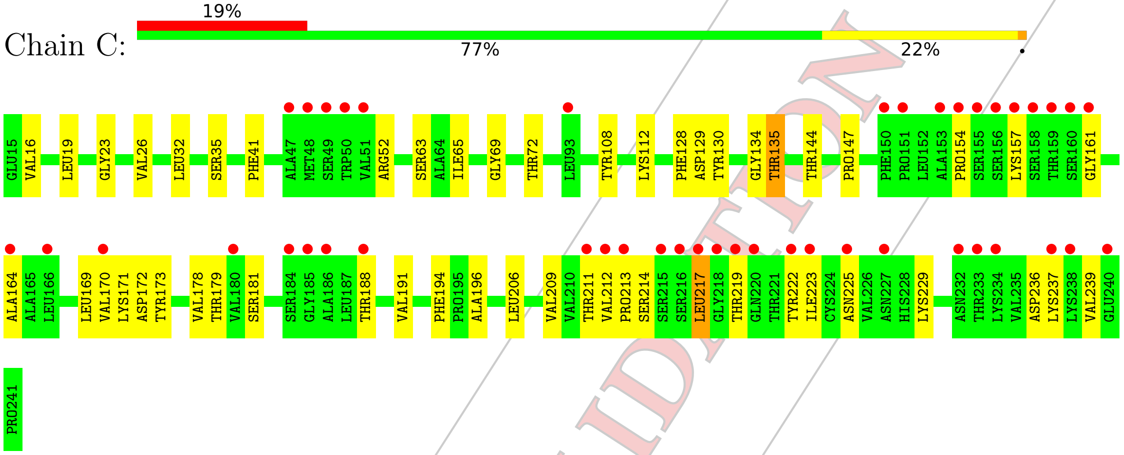

- Molecule 2:

Chain L:

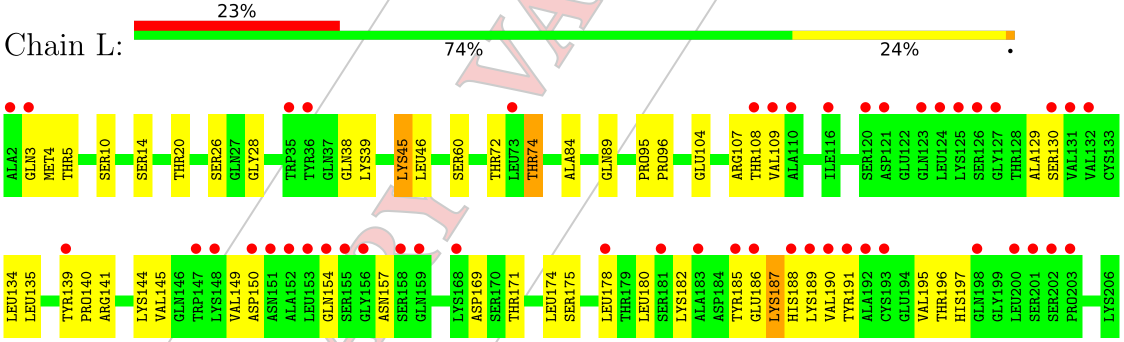

- Molecule 3:

Chain E:

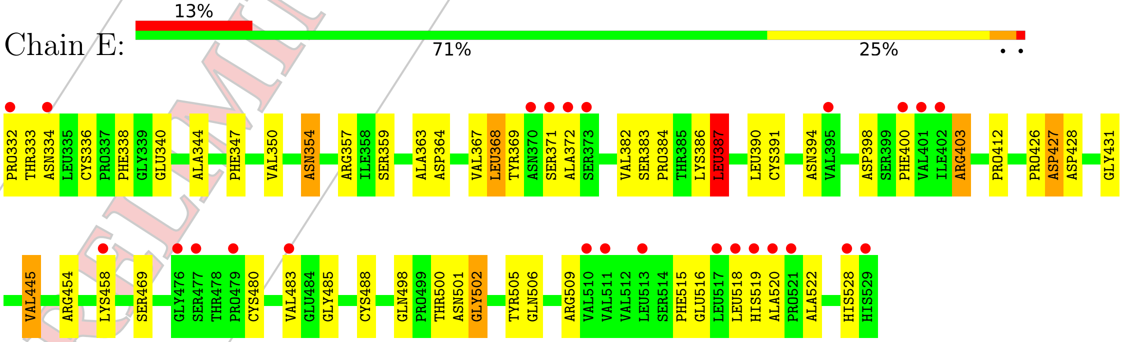

PRELIMINARY VALIDATION REPORT

4 Data and refinement statistics (i)

Property	Value	Source
Space group	H 3	Depositor
Cell constants<br>a, b, c, α, β, γ	157.19Å 157.19Å 98.32Å<br>90.00° 90.00° 120.00°	Depositor
Resolution (Å)	20.37 – 2.49<br>20.37 – 2.49	Depositor<br>EDS
% Data completeness<br>(in resolution range)	96.2 (20.37-2.49)<br>96.2 (20.37-2.49)	Depositor<br>EDS
R_{merge}	(Not available)	Depositor
R_{sym}	(Not available)	Depositor
$< I/\sigma(I) >$ [1]	1.27 (at 2.50Å)	Xtriage
Refinement program	PHENIX 1.18.2_3874	Depositor
R, R_{free}	0.214 , 0.256<br>0.214 , 0.256	Depositor<br>DCC
R_{free} test set	1436 reflections (4.72%)	wwPDB-VP
Wilson B-factor (Å^2)	50.9	Xtriage
Anisotropy	0.718	Xtriage
Bulk solvent k_{sol}(e/Å^3), B_{sol}(Å^2)	0.29 , 41.8	EDS
L-test for twinning[2]	$< \|L\| > = 0.50$, $< L^2 > = 0.34$	Xtriage
Estimated twinning fraction	0.002 for -1/3*h+1/3*k+4/3*l,-k,2/3*h+1/3*k+1/3*l<br>0.005 for -2/3*h-1/3*k-4/3*l,-1/3*h-2/3*k+4/3*l,-1/3*h+1/3*k+1/3*l<br>0.000 for -h,1/3*h-1/3*k-4/3*l,-1/3*h-2/3*k+1/3*l<br>0.012 for -1/3*h-2/3*k+4/3*l,-2/3*h-1/3*k-4/3*l,1/3*h-1/3*k-1/3*l<br>0.000 for -h,2/3*h+1/3*k+4/3*l,1/3*h+2/3*k-1/3*l<br>0.000 for 1/3*h+2/3*k-4/3*l,-k,-2/3*h-1/3*k-1/3*l<br>0.020 for h,-h-k,-l	Xtriage
F_o,F_c correlation	0.94	EDS
Total number of atoms	4835	wwPDB-VP
Average B, all atoms (Å^2)	84.0	wwPDB-VP

Xtriage's analysis on translational NCS is as follows: *The largest off-origin peak in the Patterson function is 4.16% of the height of the origin peak. No significant pseudotranslation is detected.*

[1]Intensities estimated from amplitudes.
[2]Theoretical values of $< |L| >$, $< L^2 >$ for acentric reflections are 0.5, 0.333 respectively for untwinned datasets, and 0.375, 0.2 for perfectly twinned datasets.

PRELIMINARY VALIDATION REPORT

5 Model quality (i)

5.1 Standard geometry (i)

Bond lengths and bond angles in the following residue types are not validated in this section: NAG

The Z score for a bond length (or angle) is the number of standard deviations the observed value is removed from the expected value. A bond length (or angle) with $|Z| > 5$ is considered an outlier worth inspection. RMSZ is the root-mean-square of all Z scores of the bond lengths (or angles).

Mol	Chain	Bond lengths		Bond angles	
		RMSZ	$\#\|Z\| > 5$	RMSZ	$\#\|Z\| > 5$
1	C	0.48	0/1733	0.65	1/2361 (0.0%)
2	L	0.41	0/1596	0.56	0/2170
3	E	0.50	0/1617	0.70	4/2202 (0.2%)
All	All	0.47	0/4946	0.64	5/6733 (0.1%)

Chiral center outliers are detected by calculating the chiral volume of a chiral center and verifying if the center is modelled as a planar moiety or with the opposite hand.A planarity outlier is detected by checking planarity of atoms in a peptide group, atoms in a mainchain group or atoms of a sidechain that are expected to be planar.

Mol	Chain	#Chirality outliers	#Planarity outliers
3	E	0	2

There are no bond length outliers.

All (5) bond angle outliers are listed below:

Mol	Chain	Res	Type	Atoms	Z	Observed(^{o})	Ideal(^{o})
3	E	387	LEU	CA-CB-CG	7.80	133.24	115.30
3	E	501	ASN	C-N-CA	-6.09	109.51	122.30
1	C	52	ARG	NE-CZ-NH2	-5.66	117.47	120.30
3	E	403	ARG	NE-CZ-NH1	-5.10	117.75	120.30
3	E	445	VAL	C-N-CA	-5.02	111.77	122.30

There are no chirality outliers.

All (2) planarity outliers are listed below:

Mol	Chain	Res	Type	Group
3	E	372	ALA	Peptide
3	E	502	GLY	Peptide

PRELIMINARY VALIDATION REPORT

5.2 Too-close contacts (i)

In the following table, the Non-H and H(model) columns list the number of non-hydrogen atoms and hydrogen atoms in the chain respectively. The H(added) column lists the number of hydrogen atoms added and optimized by MolProbity. The Clashes column lists the number of clashes within the asymmetric unit, whereas Symm-Clashes lists symmetry-related clashes.

Mol	Chain	Non-H	H(model)	H(added)	Clashes	Symm-Clashes
1	C	1691	0	1651	25	0
2	L	1560	0	1517	32	0
3	E	1570	0	1481	28	0
4	E	14	0	13	1	0
All	All	4835	0	4662	81	0

The all-atom clashscore is defined as the number of clashes found per 1000 atoms (including hydrogen atoms). The all-atom clashscore for this structure is 9.

All (81) close contacts within the same asymmetric unit are listed below, sorted by their clash magnitude.

Atom-1	Atom-2	Interatomic distance (Å)	Clash overlap (Å)
3:E:403:ARG:HG2	3:E:505:TYR:HA	1.64	0.79
2:L:3:GLN:HA	2:L:96:PRO:HD2	1.65	0.78
3:E:485:GLY:H	3:E:488:CYS:HB2	1.49	0.76
1:C:213:PRO:HD2	1:C:217:LEU:HD22	1.71	0.72
2:L:144:LYS:HB2	2:L:196:THR:HB	1.74	0.68
2:L:107:ARG:NH1	2:L:108:THR:O	2.26	0.68
1:C:164:ALA:HB2	1:C:214:SER:HB3	1.78	0.66
2:L:182:LYS:HA	2:L:185:TYR:HB3	1.81	0.62
1:C:196:ALA:HB2	1:C:206:LEU:HD23	1.83	0.61
1:C:23:GLY:H	1:C:135:THR:HG21	1.67	0.60
2:L:145:VAL:HG12	2:L:195:VAL:HG22	1.84	0.59
1:C:196:ALA:HA	1:C:206:LEU:HB3	1.84	0.59
3:E:502:GLY:H	3:E:506:GLN:HE21	1.53	0.57
2:L:20:THR:HG22	2:L:74:THR:HB	1.86	0.56
2:L:154:GLN:HE21	2:L:157:ASN:HD21	1.51	0.56
3:E:485:GLY:N	3:E:488:CYS:HB2	2.20	0.56
2:L:4:MET:HG2	2:L:95:PRO:HB3	1.87	0.56
2:L:28:GLY:HA2	3:E:500:THR:HG21	1.88	0.54
1:C:65:ILE:HG13	1:C:72:THR:HG22	1.89	0.54
3:E:371:SER:OG	4:E:927:NAG:H83	2.07	0.54
2:L:107:ARG:HD3	2:L:139:TYR:HB2	1.89	0.54
2:L:39:LYS:HD3	2:L:84:ALA:HB2	1.90	0.53
1:C:223:ILE:HD12	1:C:236:ASP:HB3	1.89	0.53

Continued on next page...

PRELIMINARY VALIDATION REPORT

Continued from previous page...

Atom-1	Atom-2	Interatomic distance (Å)	Clash overlap (Å)
2:L:135:LEU:HD11	2:L:145:VAL:HG11	1.90	0.52
3:E:340:GLU:O	3:E:344:ALA:HB2	2.08	0.52
1:C:154:PRO:HG3	1:C:239:VAL:HG12	1.89	0.52
3:E:391:CYS:HB3	3:E:522:ALA:HB1	1.91	0.52
1:C:170:VAL:HG11	1:C:178:VAL:HG11	1.91	0.52
1:C:108:TYR:O	1:C:134:GLY:HA2	2.11	0.51
1:C:181:SER:HB2	1:C:225:ASN:HB2	1.93	0.50
3:E:364:ASP:O	3:E:367:VAL:HG22	2.12	0.49
3:E:480:CYS:O	3:E:483:VAL:HG22	2.13	0.49
2:L:130:SER:HA	2:L:178:LEU:O	2.13	0.49
1:C:147:PRO:HB3	1:C:173:TYR:HB3	1.94	0.48
1:C:41:PHE:CE2	1:C:112:LYS:HD2	2.48	0.48
2:L:150:ASP:HA	2:L:190:VAL:HB	1.95	0.48
3:E:383:SER:HB3	3:E:386:LYS:HD3	1.94	0.48
1:C:161:GLY:O	1:C:214:SER:OG	2.30	0.48
1:C:169:LEU:HD23	1:C:171:LYS:HB2	1.96	0.47
3:E:336:CYS:SG	3:E:363:ALA:HB2	2.54	0.47
2:L:144:LYS:O	2:L:195:VAL:HA	2.14	0.47
2:L:149:VAL:HG13	2:L:191:TYR:HE1	1.80	0.47
2:L:45:LYS:HA	2:L:45:LYS:HD2	1.34	0.46
3:E:518:LEU:O	3:E:520:ALA:N	2.49	0.46
2:L:129:ALA:N	2:L:180:LEU:O	2.41	0.46
1:C:194:PHE:CZ	2:L:175:SER:HB3	2.52	0.45
1:C:171:LYS:HG2	1:C:172:ASP:OD2	2.17	0.45
3:E:364:ASP:OD1	3:E:367:VAL:HG13	2.17	0.45
1:C:65:ILE:HD11	1:C:69:GLY:HA2	2.00	0.44
1:C:112:LYS:O	1:C:128:PHE:HA	2.18	0.44
1:C:212:VAL:HG21	1:C:222:TYR:OH	2.18	0.44
3:E:350:VAL:HA	3:E:400:PHE:HB2	1.99	0.44
2:L:169:ASP:OD1	2:L:171:THR:OG1	2.23	0.44
3:E:431:GLY:HA2	3:E:515:PHE:CD2	2.53	0.44
2:L:187:LYS:HB3	2:L:187:LYS:HE3	1.72	0.44
3:E:354:ASN:O	3:E:398:ASP:HA	2.18	0.44
1:C:209:VAL:HG21	2:L:134:LEU:HD13	1.99	0.44
2:L:45:LYS:HE2	2:L:46:LEU:H	1.83	0.43
3:E:427:ASP:OD1	3:E:427:ASP:N	2.51	0.43
1:C:129:ASP:OD1	1:C:130:TYR:N	2.50	0.43
1:C:188:THR:O	1:C:191:VAL:HG22	2.19	0.43
2:L:139:TYR:HB3	2:L:140:PRO:HD3	2.01	0.43
2:L:189:LYS:HD3	2:L:189:LYS:HA	1.86	0.42
1:C:16:VAL:HB	1:C:130:TYR:CE2	2.54	0.42

Continued on next page...

PRELIMINARY VALIDATION REPORT

Continued from previous page...

Atom-1	Atom-2	Interatomic distance (Å)	Clash overlap (Å)
3:E:332:PRO:HB2	3:E:333:THR:H	1.68	0.42
3:E:412:PRO:HB3	3:E:426:PRO:O	2.20	0.42
1:C:171:LYS:NZ	2:L:130:SER:HB3	2.34	0.42
2:L:38:GLN:O	2:L:84:ALA:HB1	2.19	0.42
2:L:135:LEU:HD13	2:L:174:LEU:HD22	2.02	0.42
3:E:458:LYS:HA	3:E:458:LYS:HD2	1.89	0.42
2:L:140:PRO:HD2	2:L:197:HIS:HE1	1.85	0.41
3:E:347:PHE:CD2	3:E:509:ARG:HG2	2.54	0.41
2:L:169:ASP:O	2:L:171:THR:HG23	2.21	0.41
3:E:454:ARG:NH2	3:E:469:SER:O	2.51	0.41
3:E:502:GLY:N	3:E:506:GLN:HE21	2.17	0.41
3:E:368:LEU:HA	3:E:368:LEU:HD23	1.77	0.41
2:L:135:LEU:HB2	2:L:174:LEU:HB3	2.03	0.41
3:E:338:PHE:HE2	3:E:363:ALA:HB1	1.86	0.41
3:E:394:ASN:HB2	3:E:516:GLU:OE1	2.20	0.41
3:E:384:PRO:HA	3:E:387:LEU:CD2	2.51	0.40
2:L:140:PRO:HD2	2:L:197:HIS:CE1	2.57	0.40

There are no symmetry-related clashes.

5.3 Torsion angles (i)

5.3.1 Protein backbone (i)

In the following table, the Percentiles column shows the percent Ramachandran outliers of the chain as a percentile score with respect to all X-ray entries followed by that with respect to entries of similar resolution.

The Analysed column shows the number of residues for which the backbone conformation was analysed, and the total number of residues.

Mol	Chain	Analysed	Favoured	Allowed	Outliers	Percentiles	
1	C	225/227 (99%)	211 (94%)	14 (6%)	0	100	100
2	L	203/205 (99%)	190 (94%)	13 (6%)	0	100	100
3	E	196/198 (99%)	180 (92%)	16 (8%)	0	100	100
All	All	624/630 (99%)	581 (93%)	43 (7%)	0	100	100

There are no Ramachandran outliers to report.

PRELIMINARY VALIDATION REPORT

5.3.2 Protein sidechains (i)

In the following table, the Percentiles column shows the percent sidechain outliers of the chain as a percentile score with respect to all X-ray entries followed by that with respect to entries of similar resolution.

The Analysed column shows the number of residues for which the sidechain conformation was analysed, and the total number of residues.

Mol	Chain	Analysed	Rotameric	Outliers	Percentiles	
1	C	187/187 (100%)	173 (92%)	14 (8%)	13	26
2	L	176/176 (100%)	161 (92%)	15 (8%)	10	21
3	E	171/171 (100%)	156 (91%)	15 (9%)	10	19
All	All	534/534 (100%)	490 (92%)	44 (8%)	11	22

All (44) residues with a non-rotameric sidechain are listed below:

Mol	Chain	Res	Type
1	C	19	LEU
1	C	26	VAL
1	C	32	LEU
1	C	35	SER
1	C	63	SER
1	C	135	THR
1	C	144	THR
1	C	157	LYS
1	C	179	THR
1	C	211	THR
1	C	217	LEU
1	C	219	THR
1	C	229	LYS
1	C	237	LYS
2	L	5	THR
2	L	10	SER
2	L	14	SER
2	L	26	SER
2	L	45	LYS
2	L	60	SER
2	L	72	THR
2	L	74	THR
2	L	89	GLN
2	L	104	GLU
2	L	109	VAL
2	L	141	ARG

Continued on next page...

PRELIMINARY VALIDATION REPORT

Continued from previous page...

Mol	Chain	Res	Type
2	L	186	GLU
2	L	187	LYS
2	L	188	HIS
3	E	334	ASN
3	E	354	ASN
3	E	357	ARG
3	E	359	SER
3	E	368	LEU
3	E	369	TYR
3	E	382	VAL
3	E	387	LEU
3	E	390	LEU
3	E	427	ASP
3	E	428	ASP
3	E	445	VAL
3	E	498	GLN
3	E	519	HIS
3	E	528	HIS

Sometimes sidechains can be flipped to improve hydrogen bonding and reduce clashes. All (1) such sidechains are listed below:

Mol	Chain	Res	Type
2	L	154	GLN

5.3.3 RNA (i)

There are no RNA molecules in this entry.

5.4 Non-standard residues in protein, DNA, RNA chains (i)

There are no non-standard protein/DNA/RNA residues in this entry.

5.5 Carbohydrates (i)

There are no monosaccharides in this entry.

5.6 Ligand geometry (i)

1 ligand is modelled in this entry.

PRELIMINARY VALIDATION REPORT

In the following table, the Counts columns list the number of bonds (or angles) for which Mogul statistics could be retrieved, the number of bonds (or angles) that are observed in the model and the number of bonds (or angles) that are defined in the Chemical Component Dictionary. The Link column lists molecule types, if any, to which the group is linked. The Z score for a bond length (or angle) is the number of standard deviations the observed value is removed from the expected value. A bond length (or angle) with $|Z| > 2$ is considered an outlier worth inspection. RMSZ is the root-mean-square of all Z scores of the bond lengths (or angles).

Mol	Type	Chain	Res	Link	Bond lengths			Bond angles		
					Counts	RMSZ	$\#|Z| > 2$	Counts	RMSZ	$\#|Z| > 2$
4	NAG	E	927	3	14,14,15	1.22	1 (7%)	17,19,21	1.48	1 (5%)

In the following table, the Chirals column lists the number of chiral outliers, the number of chiral centers analysed, the number of these observed in the model and the number defined in the Chemical Component Dictionary. Similar counts are reported in the Torsion and Rings columns. '-' means no outliers of that kind were identified.

Mol	Type	Chain	Res	Link	Chirals	Torsions	Rings
4	NAG	E	927	3	-	2/6/23/26	0/1/1/1

All (1) bond length outliers are listed below:

Mol	Chain	Res	Type	Atoms	Z	Observed(Å)	Ideal(Å)
4	E	927	NAG	O5-C1	4.35	1.50	1.43

All (1) bond angle outliers are listed below:

Mol	Chain	Res	Type	Atoms	Z	Observed(^{o})	Ideal(^{o})
4	E	927	NAG	C1-O5-C5	5.33	119.41	112.19

There are no chirality outliers.

All (2) torsion outliers are listed below:

Mol	Chain	Res	Type	Atoms
4	E	927	NAG	O5-C5-C6-O6
4	E	927	NAG	C4-C5-C6-O6

There are no ring outliers.

1 monomer is involved in 1 short contact:

Mol	Chain	Res	Type	Clashes	Symm-Clashes
4	E	927	NAG	1	0

PRELIMINARY VALIDATION REPORT

5.7 Other polymers (i)

There are no such residues in this entry.

5.8 Polymer linkage issues (i)

There are no chain breaks in this entry.

PRELIMINARY VALIDATION REPORT

6 Fit of model and data (i)

6.1 Protein, DNA and RNA chains (i)

In the following table, the column labelled '#RSRZ> 2' contains the number (and percentage) of RSRZ outliers, followed by percent RSRZ outliers for the chain as percentile scores relative to all X-ray entries and entries of similar resolution. The OWAB column contains the minimum, median, 95th percentile and maximum values of the occupancy-weighted average B-factor per residue. The column labelled 'Q< 0.9' lists the number of (and percentage) of residues with an average occupancy less than 0.9.

Mol	Chain	Analysed	<RSRZ>	#RSRZ>2			OWAB(Å^2)	Q<0.9
1	C	227/227 (100%)	1.04	44 (19%)	1	1	38, 53, 189, 234	0
2	L	205/205 (100%)	1.06	48 (23%)	0	0	43, 91, 158, 187	0
3	E	198/198 (100%)	0.37	25 (12%)	3	3	39, 60, 105, 132	0
All	All	630/630 (100%)	0.84	117 (18%)	1	1	38, 68, 161, 234	0

All (117) RSRZ outliers are listed below:

Mol	Chain	Res	Type	RSRZ
1	C	217	LEU	19.8
2	L	192	ALA	11.7
1	C	155	SER	11.6
1	C	158	SER	9.2
1	C	154	PRO	9.2
1	C	156	SER	9.0
1	C	161	GLY	8.8
1	C	188	THR	6.9
2	L	153	LEU	6.7
1	C	213	PRO	6.3
1	C	153	ALA	5.8
2	L	186	GLU	5.6
1	C	186	ALA	5.6
1	C	218	GLY	5.4
2	L	130	SER	5.3
1	C	160	SER	5.1
2	L	198	GLN	5.1
1	C	157	LYS	4.9
2	L	155	SER	4.7
1	C	219	THR	4.7
2	L	151	ASN	4.5
2	L	191	TYR	4.4
2	L	121	ASP	4.4

Continued on next page...

PRELIMINARY VALIDATION REPORT

Continued from previous page...

Mol	Chain	Res	Type	RSRZ
2	L	154	GLN	4.3
2	L	125	LYS	4.2
1	C	225	ASN	4.2
1	C	238	LYS	4.2
3	E	476	GLY	4.1
1	C	216	SER	4.0
1	C	185	GLY	4.0
2	L	156	GLY	3.9
2	L	123	GLN	3.9
1	C	232	ASN	3.8
2	L	189	LYS	3.8
2	L	201	SER	3.8
1	C	159	THR	3.8
1	C	50	TRP	3.7
3	E	521	PRO	3.5
2	L	124	LEU	3.5
3	E	518	LEU	3.5
2	L	150	ASP	3.5
2	L	185	TYR	3.4
2	L	148	LYS	3.4
2	L	181	SER	3.3
2	L	190	VAL	3.3
2	L	152	ALA	3.3
3	E	371	SER	3.3
3	E	513	LEU	3.2
1	C	222	TYR	3.2
3	E	520	ALA	3.2
1	C	49	SER	3.2
2	L	73	LEU	3.2
2	L	116	ILE	3.1
2	L	120	SER	3.1
2	L	35	TRP	3.0
2	L	202	SER	3.0
3	E	529	HIS	2.9
3	E	373	SER	2.9
2	L	158	SER	2.9
2	L	2	ALA	2.9
1	C	184	SER	2.8
3	E	402	ILE	2.8
2	L	188	HIS	2.8
2	L	127	GLY	2.8
2	L	168	LYS	2.8

Continued on next page...

PRELIMINARY VALIDATION REPORT

Continued from previous page...

Mol	Chain	Res	Type	RSRZ
1	C	48	MET	2.8
3	E	477	SER	2.7
1	C	215	SER	2.7
3	E	510	VAL	2.7
1	C	150	PHE	2.7
2	L	139	TYR	2.6
2	L	183	ALA	2.6
3	E	528	HIS	2.6
1	C	93	LEU	2.6
1	C	51	VAL	2.6
2	L	147	TRP	2.6
2	L	109	VAL	2.6
2	L	3	GLN	2.5
3	E	334	ASN	2.5
3	E	372	ALA	2.5
1	C	170	VAL	2.5
3	E	401	VAL	2.5
1	C	220	GLN	2.4
1	C	212	VAL	2.4
1	C	237	LYS	2.4
2	L	110	ALA	2.4
2	L	36	TYR	2.4
3	E	479	PRO	2.4
1	C	151	PRO	2.4
2	L	203	PRO	2.4
1	C	227	ASN	2.3
1	C	234	LYS	2.3
1	C	223	ILE	2.3
1	C	233	THR	2.3
2	L	193	CYS	2.3
1	C	240	GLU	2.3
1	C	47	ALA	2.3
3	E	370	ASN	2.2
3	E	395	VAL	2.2
3	E	332	PRO	2.2
2	L	159	GLN	2.2
3	E	483	VAL	2.2
2	L	178	LEU	2.2
3	E	511	VAL	2.2
2	L	200	LEU	2.2
2	L	108	THR	2.1
3	E	458	LYS	2.1

Continued on next page...

PRELIMINARY VALIDATION REPORT

Continued from previous page...

Mol	Chain	Res	Type	RSRZ
1	C	164	ALA	2.1
1	C	211	THR	2.1
2	L	131	VAL	2.1
1	C	180	VAL	2.1
3	E	517	LEU	2.1
3	E	519	HIS	2.0
2	L	126	SER	2.0
1	C	166	LEU	2.0
2	L	132	VAL	2.0
3	E	400	PHE	2.0

6.2 Non-standard residues in protein, DNA, RNA chains (i)

There are no non-standard protein/DNA/RNA residues in this entry.

6.3 Carbohydrates (i)

There are no monosaccharides in this entry.

6.4 Ligands (i)

In the following table, the Atoms column lists the number of modelled atoms in the group and the number defined in the chemical component dictionary. The B-factors column lists the minimum, median, 95th percentile and maximum values of B factors of atoms in the group. The column labelled 'Q< 0.9' lists the number of atoms with occupancy less than 0.9.

Mol	Type	Chain	Res	Atoms	RSCC	RSR	B-factors($Å^2$)	Q<0.9
4	NAG	E	927	14/?	0.79	0.17	71,74,84,85	0

6.5 Other polymers (i)

There are no such residues in this entry.

PRELIMINARY VALIDATION REPORT
