# Supplementary material for: Etesevimab in combination with JS026 neutralizing SARS-CoV-2 and its variants
Source: Emerg Microbes Infect. 2022 Feb 10;11(1):548–51. doi: 10.1080/22221751.2022.2032374 (PMC8843163; doi:10.1080/22221751.2022.2032374)
Supplement: Supplemental Material [file TEMI_A_2032374_SM2670.zip › Suppl files/Revised_supplemental_materials_clean.docx]

**Etesevimab in combination with JS026 neutralizing SARS-CoV-2 and its variants**

Fengze Wang^1,2,6^, Li Li^3,6^, Yang Dou^4,6^, Rui Shi^1^, Xiaomin Duan^1,2^, Hongchuan Liu^3^, Jing Zhang^3^, DanDan Liu^3^, Jing Wu^3^, Yang He^3^, Jun Lan^5^, Bai Lu^4*^, Hui Feng^3*^, and Jinghua Yan^1,2*^

^1^CAS Key Laboratory of Pathogenic Microbiology and Immunology, Institute of Microbiology, Chinese Academy of Sciences, Beijing 100101, China.

^2^University of Chinese Academy of Sciences, Beijing 100049, China.

^3^Shanghai Junshi Biosciences Co. Ltd, Shanghai 200126, China.

^4^School of Pharmaceutical Sciences, IDG/McGovern Institute for Brain Research, Tsinghua University, Beijing, 100084, China.

^5^Beijing Advanced Innovation Center for Structural Biology, School of Life Sciences, Tsinghua University, Beijing, 100084, China.

^6^These authors contributed equally.

^*^Correspondence: bai_lu@tsinghua.edu.cn (B.L.), hui_feng@junshipharma.com (H.F.), and yanjh@im.ac.cn (J.Y.)

**Keywords: SARS-CoV-2, Etesevimab, JS026, antibody cocktail, variants of concern**

**Method**

**Ethics statement**

This study was approved and issued by the Ethics Committee of the Institute of Microbiology, Chinese Academy of Sciences.

**Blocking assay**

To measure the potency of JS026 blocking the interaction between SARS-CoV-2/SARS-CoV-2 VOCs RBD and human ACE2 on the cell surface, HEK293-hACE2 (overexpressed with human ACE2, 10^5^ cells/well) were incubated with different concentrations of JS026 or negative control antibody (from 100 μg/mL to 5 ng/mL, 3-fold serial dilutions) in staining buffer (PBS containing 1% of FBS) at 4℃ for 30 min, in the presence of a fixed concentration of biotinylated recombinant RBD proteins. Biotinylated recombinant SARS-CoV-2 WT RBD (ACRO, Cat# SPD-C82E8), Alpha variant RBD (ACRO, Cat# SPD-C82E6), Beta variant RBD (ACRO, Cat# SPD-C82E5), Gamma variant RBD (ACRO, Cat# SPD-C82E7), and Delta variant B.1.617.2 RBD (ACRO, Cat#80048) were used at the concentration of 1.5 μg/mL, 0.3 μg/mL, 0.5 μg/mL, 0.5 μg/mL, 0.5 μg/mL, 0.5 μg/mL and 0.5 μg/mL, respectively. After washing the cells twice with staining buffer, cells were subsequently stained with PE Streptavidin (BioLegend, Cat#405203) at 1:100 (v/v) in staining buffer at 4°C for 30min to detect cell-bound RBD proteins. Samples were acquired on the flow cytometer (BD FACS Canto Ⅱ). Mean Fluorescence Intensity (MFI) of bound RBD proteins was analyzed and IC50 were determined using a log (inhibitor) vs response-variable slope curve fit by GraphPad Prism.

**BLI**

The Octet RED384 system (Sartorius Fortebio) was used to study the kinetics of SARS-CoV-2 neutralizing antibodies binding to WT and VOCs RBD proteins. The assays were performed in solid black 96-well plates (Greiner Bio-One, Cat. No. 655209) using protein A biosensors (Sartorius Fortebio, Cat. No. 18-5010). All reagents were diluted in assay buffer (PBS containing 0.02% Tween-20). Briefly, 1 μg/mL of neutralizing antibody was loaded onto biosensors, which were then dipped into RBD solutions from different mutants using a series of respective gradient concentrations (100 nM, 50 nM, 25 nM, 12.5 nM, and 6.25 nM). Then biosensors were dipped into assay buffer wells for dissociation. The KD was determined using a 1:1 binding kinetics model and was analyzed with global fitting using Octet RED384 Data Analysis 12.0.

The competitive binding of JS026 was performed at 25 °C in a buffer containing 137 mM NaCl, 2.7 mM KCl, 10 mM Na_2_HPO_4_, 2 mM KH_2_PO_4_, 0.005% Tween-20, and 50 mM Tris-HCl, pH7.4. Streptavidin biosensors were pre-equilibrated in the buffer for 10 min. Biotinylated SARS-CoV-2 RBD was loaded onto streptavidin biosensors. Then, SARS-CoV-2 RBD was sequential flowed JS026 protein and another mAb. The interference patterns from the biotinylated SARS-CoV-2 RBD with buffer or mAbs protein were analysed as two sets of controls.

**Neutralization assay**

In the pseudovirus neutralization assay, various concentrations of JS026, etesevimab, cocktail, and negative control antibody (from 100 μg/mL to 0.1 pg/mL, 10-fold serial dilutions) were pre-incubated in DMEM medium (DMEM+10% FBS) with different virus titer of WT or SARS-CoV-2 VOCs pseudovirus (Beijing SanYao Science & Technology Development) at 37°C for 1h, respectively. Dosages of WT SARA-CoV-2 (Cat#80033), Alpha variant (Cat#80043), Beta variant (Cat#80044), Gamma variant (Cat#80045), and Delta variant (Cat#80048) pseudovirus were 2 μL/well, respectively. Then the pseudovirus-antibody mixture was co-cultured with HEK293-hACE2 cells in the 96-well white plate (Corning, Cat#3917) at a CO_2_ incubator overnight. On the second day, 50 μL of Bright-Lite Luciferase substrate (Vazyme, Cat# DD1204-03) was added to each well and the plate was incubated at room temperature for 5 min protected from light. Luciferase signal (RLU value) was measured using a Microplate Reader (SpectraMax M5). IC50 values of test articles were calculated using GraphPad Prism software and non-linear regression of log (inhibitor) vs response-variable slope was applied.

An authentic SARS-CoV-2 neutralization assay was performed in Biosafety Level-3 facility. Brieﬂy, serial 2-fold dilutions of 50 µL antibody proteins were mixed with an equal volume of SARS-CoV-2 working stock containing 100 TCID50 and incubated at 37°C for 1 h. The mixture was added into an equal volume of conﬂuent Vero E6 cells with 8 repeats and incubated at 37°C in a CO_2_ incubator for 3 days. Cytopathic effect (CPE) in each well was observed and recorded on day 3. The ND50 was calculated using Prism GraphPad 8.0.

**Animal experiments**

Eight hACE2 transgenic mice (HACE2-KI/NIFDC 8-10-week-old female mice from National Institutes for Food and Drug Control) in the prophylactic group received JS026 (20 mg/kg). One day post-dosing, mice were infected with 5×10^5^ TCID50 of SARS-CoV-2 via the intranasal infection. Other mice injected JS026 (20 mg/kg), cocktail (10 mg/kg etesevimab and 10 mg/kg JS026), or PBS. All mice were euthanized on the 5th day following the challenge. Five mice in each group were harvested for virus load detection and three mice for pathological examination.

Lung tissues of challenged mice were homogenized and extracted viral RNA by magnetic bead extraction Kit (EmerTher). OFR1ab-F: 5’-CCCTGTGGGTTTTACACTTAA-3’, OFR1ab-R: 5’-ACGATTGTGCATCAGCTGA-3’, Probe-ORF1ab: 5’-the FAM-CCGTCTGCGGTATGTGGAAAGGTTATGG-BHQ1-3’ targeting ORF1ab gene were used to detect viral RNA. The amplification was performed as followed: 42℃ for 5 minutes, 95℃ for 10 seconds followed by 40 cycles consisting of 95℃ for 3 seconds, 60℃ for 30 seconds, and a default melting curve step in an Applied Biosystems QuantStudio 5 Real-Time PCR System. The limit of detection is 40 RNA copies per reaction mixture.

For histopathology analysis, lung samples were collected and immobilized in 10% neutral buffer formaldehyde and embedded in paraffin wax. Tissue sections were treated with hematoxylin and eosin (H&E) and analyzed microscopically.

**Crystallization and Structure solution**

The purified SARS-CoV-2 RBD was mixed and incubated with the cleaved Fab of JS026 (with a molar ratio of 1.0: 1.2). RBD/JS026-Fab was further purified by gel-filtration chromatography. Crystals were successfully obtained in 2% v/v Tacsimate pH5.0, 0.1 M Sodium citrate tribasic dihydrate pH5.6, 16% w/v Polyethylene glycol 3,350. Diffraction data were collected at 100 K and a wavelength of 1.071 Å on the BL17U1 beam line of the Shanghai Synchrotron Research Facility (SSRF). Diffraction data was auto-processed with aquarium pipeline and the data processing statistics are listed in Supplementary Table 2. The structure was solved by the molecular replacement method. Subsequent model building and refinement were performed using COOT and PHENIX, respectively. The structure has been deposited in the PDB under accession number 7F7E.


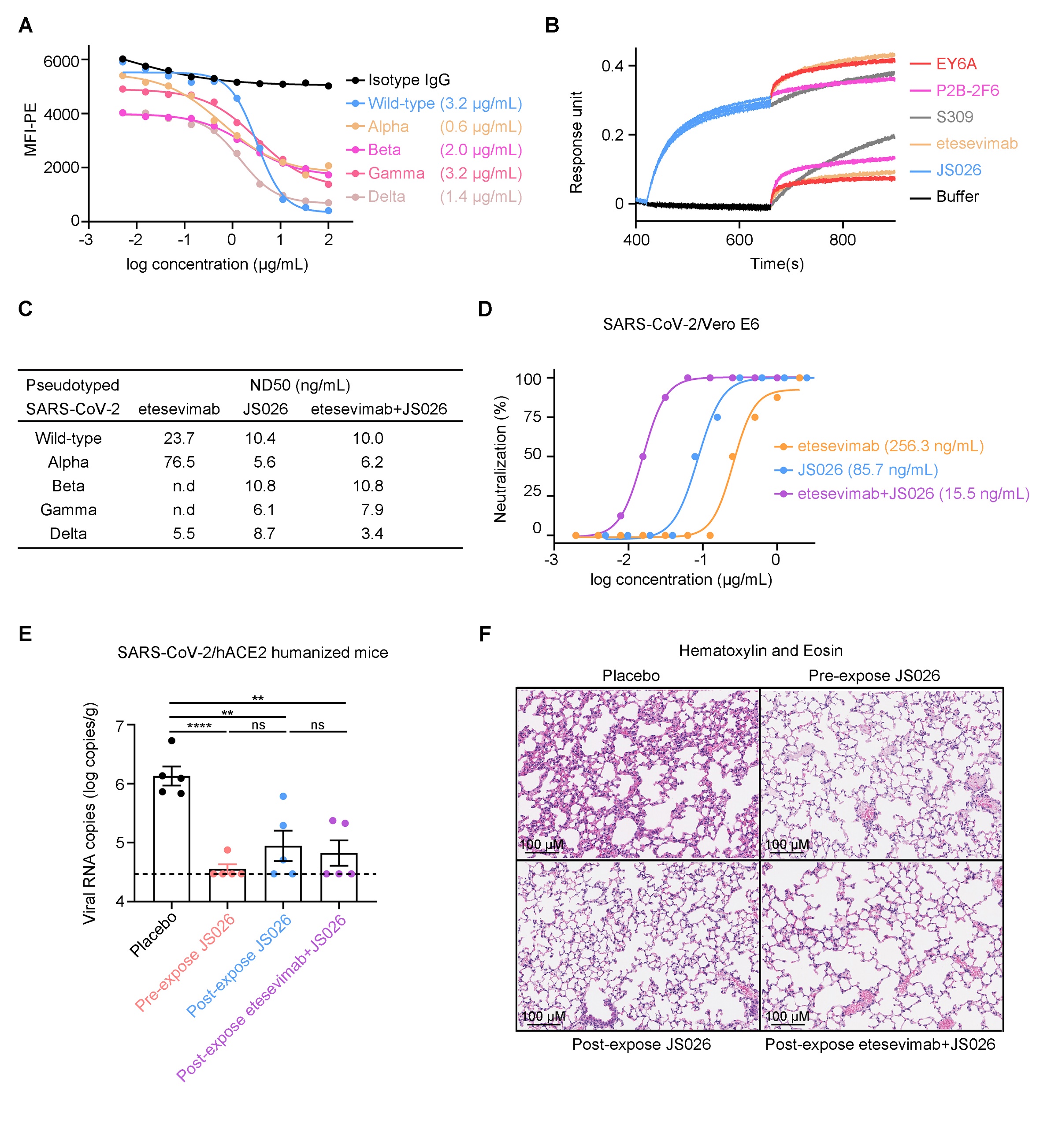
**Supplementary Figure 1 Epitope mapping of JS026 on SARS-CoV-2-RBD.** Immobilized RBD proteins were first saturated with JS026 and then exposed to one of the previously reported SARS-CoV-2-RBD-specific mAbs via BLI. The binding profiles are shown.


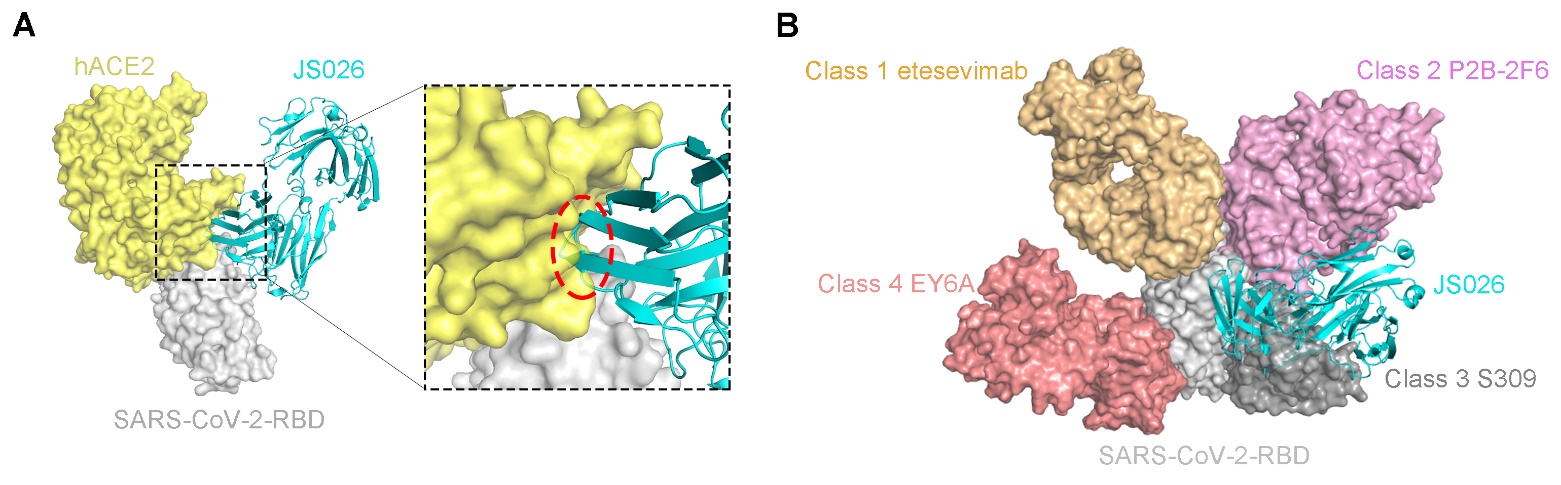
**Supplementary Figure 2 JS026: its blocking mechanism and epitopes mapping. A,** Superimposition of JS026/RBD complex and hACE2/RBD (PDB: 6LZG) reveal the steric clash between JS026 and hACE2. The JS026-Fab, hACE2, and SARS-CoV-2-RBD are colored differently as indicated. **B,** SARS-CoV-2-RBD (PDB: 6LZG) is shown in surface (gray). Epitope mapping of classes 1-4 mAbs, etesevimab (7C01), P2B-2F6 (7BWJ), S309 (6WPT), and EY6A (6ZFO), are colored in orange, pink, dark gray, and red, respectively. The JS026-Fab (7F7E) is shown in cartoon (cyan).
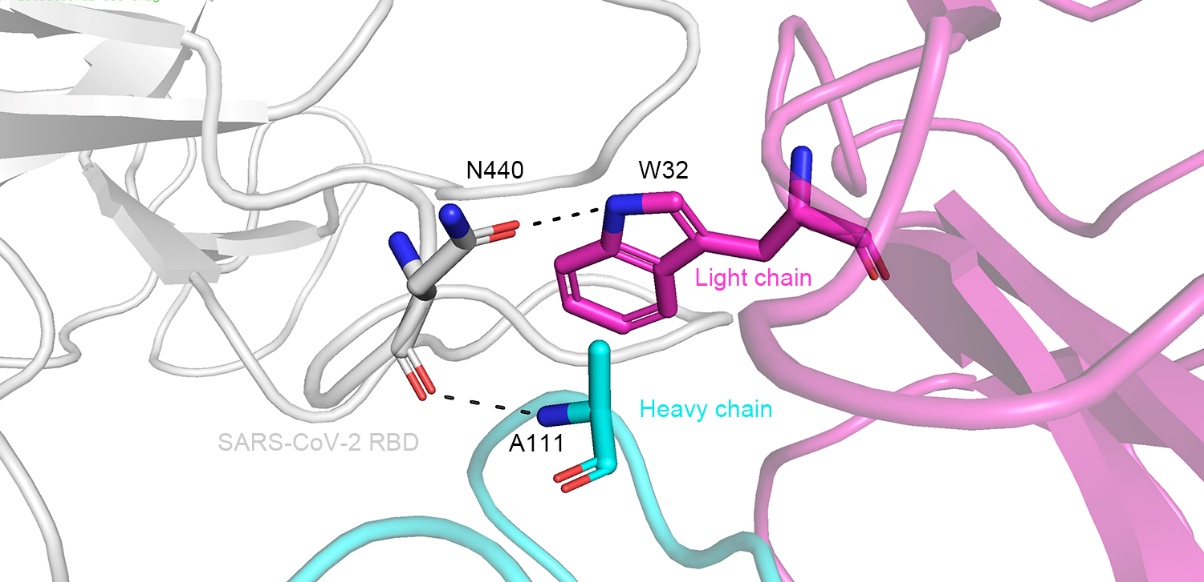
**Supplementary Figure 3 N440 of RBD forces multiple contacts with JS026.** The JS026 Fab is shown as a cartoon with a translucent surface (cyan for heavy chain and purple for light chain) and the RBD is rayed in gray. Residues involved in hydrogen bond interactions are shown as sticks, and the hydrogen bonds are shown as dashed black lines.


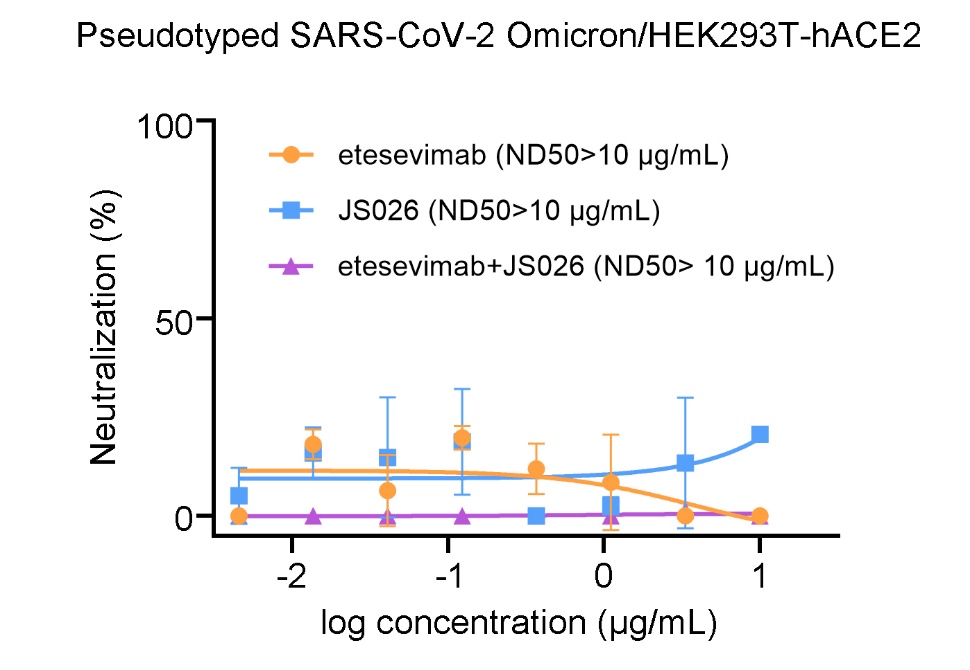
**Supplementary Figure 4 The neutralization activity of mAbs against Omicron.** The mixtures of pseudotyped Omcron were incubated with serially diluted etesevimab, JS026, or the JS026/etesevimab cocktail. The mixtures were then added to HEK293T-hACE2 cells for another incubation. One of two independent experiment data is shown.

**Supplementary Table 1 Measurement of binding affinities of JS026 to VOCs RBD proteins.**

| SARS-CoV-2-RBD | BLI | | |
| --- | --- | --- | --- |
| variants | Ka(1/Ms) | Kd(1/s) | KD(M) |
| Wild-type | 1.06E5 | 2.15E-4 | 2.04E-9 |
| Alpha | 2.43E5 | 3.16E-4 | 1.30E-9 |
| Beta | 2.01E5 | 1.76E-4 | 0.88E-9 |
| Gamma | 2.16E5 | 2.41E-4 | 1.12E-9 |
| Delta | 2.20E5 | 1.61E-4 | 0.73E-9 |

The representative data of three replicate experiments were shown.

**Supplementary Table 2 Crystallization data collection and refinement statistics.**

|  | JS026/SARS-CoV-2-RBD |
| --- | --- |
| **Data collection** |  |
| Space group | R3:H |
| Cell dimensions |  |
| *a, b, c* (Å) | 157.189, 157.189, 98.319 |
| *α, β, γ* (°) | 90, 90, 120 |
| Resolution | 50.00-2.49 (2.579-2.49)^*^ |
| Unique | 30468 (2375) |
| Completeness | 96.03 (75.67) |
| Rpim | 0.054 (0.450) |
| *I/σI* | 14.22 (1.08) |
| CC1/2 (%) | 0.995 (0.565) |
| Redundancy | 9.6 (5.9) |
|  |  |
| **Refinement** |  |
| Resolution | 20.37-2.49 |
| No. reflections | 30444 |
| *R*_work_/*R*_free_ | 0.2143/0.2562 |
| No. atoms |  |
| Protein | 4821 |
| Ligand/ion | 14 |
| Water |  |
| B-factors |  |
| Protein | 84.20 |
| Ligand/ion | 76.17 |
| Water |  |
| R.M.S. |  |
| Bond lengths | 0.008 |
| Bond angles | 1.05 |
| Ramchandran |  |
| Statistics (%) |  |
| Favored | 93.11 |
| Allowed | 6.89 |
| Disallowed | 0 |

Diffraction data from one crystal were used for structure determination.

^*^Values in parentheses are for the highest-resolution shell.
