# Supplementary figures and images for: Etesevimab in combination with JS026 neutralizing SARS-CoV-2 and its variants
Source: Emerg Microbes Infect. 2022 Feb 10;11(1):548–51. doi: 10.1080/22221751.2022.2032374 (PMC8843163; doi:10.1080/22221751.2022.2032374)

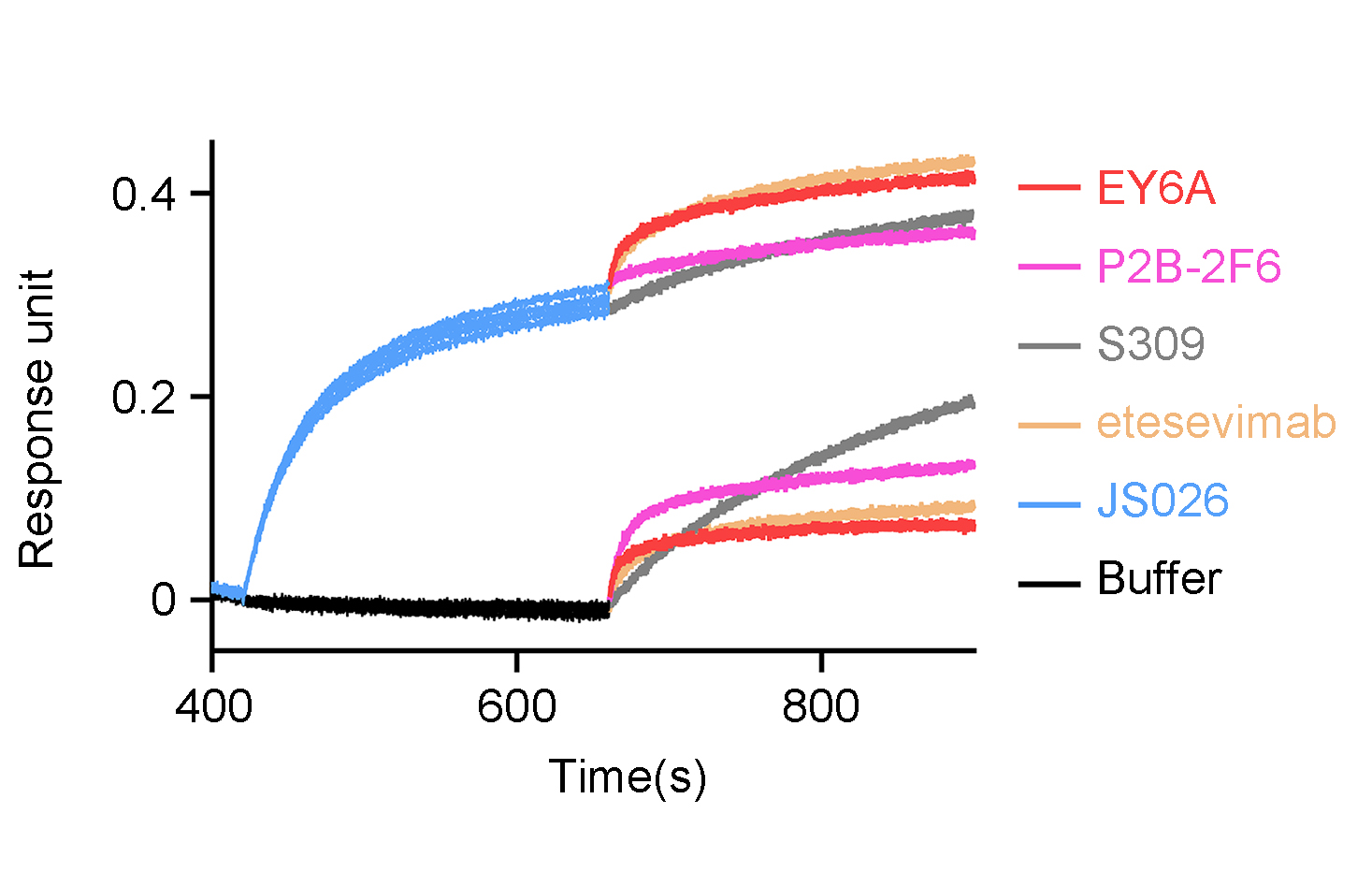

Supplement: Supplemental Material [file TEMI_A_2032374_SM2670.zip › Suppl files/Supplementary Figure 1.jpg]

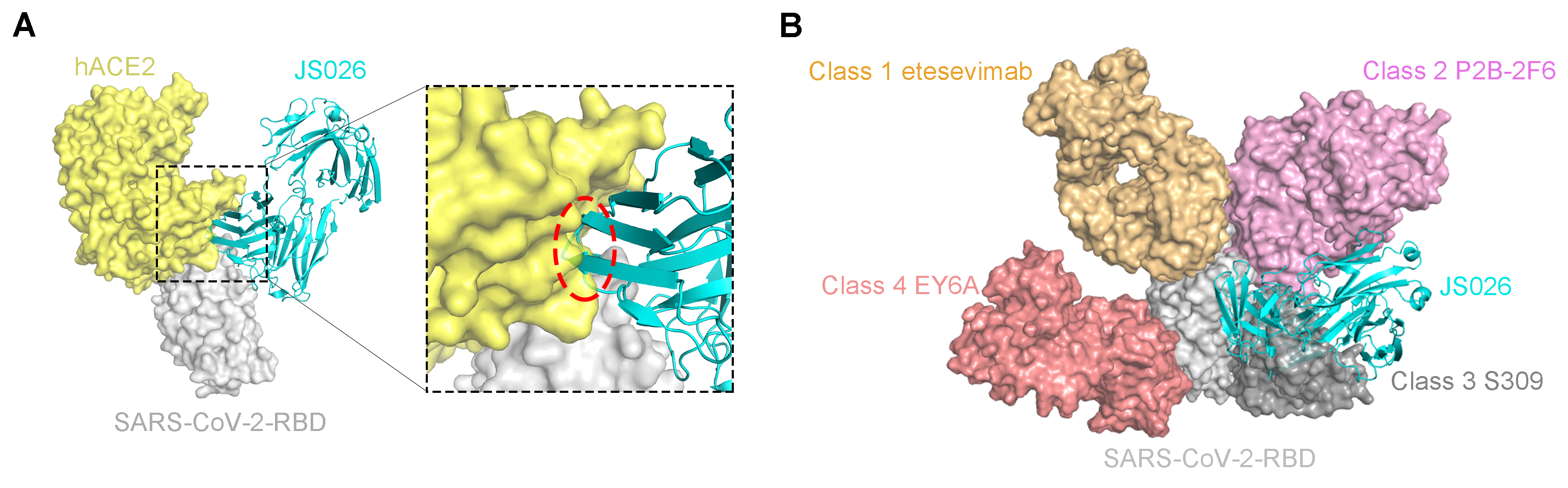

Supplement: Supplemental Material [file TEMI_A_2032374_SM2670.zip › Suppl files/Supplementary Figure 2.jpg]

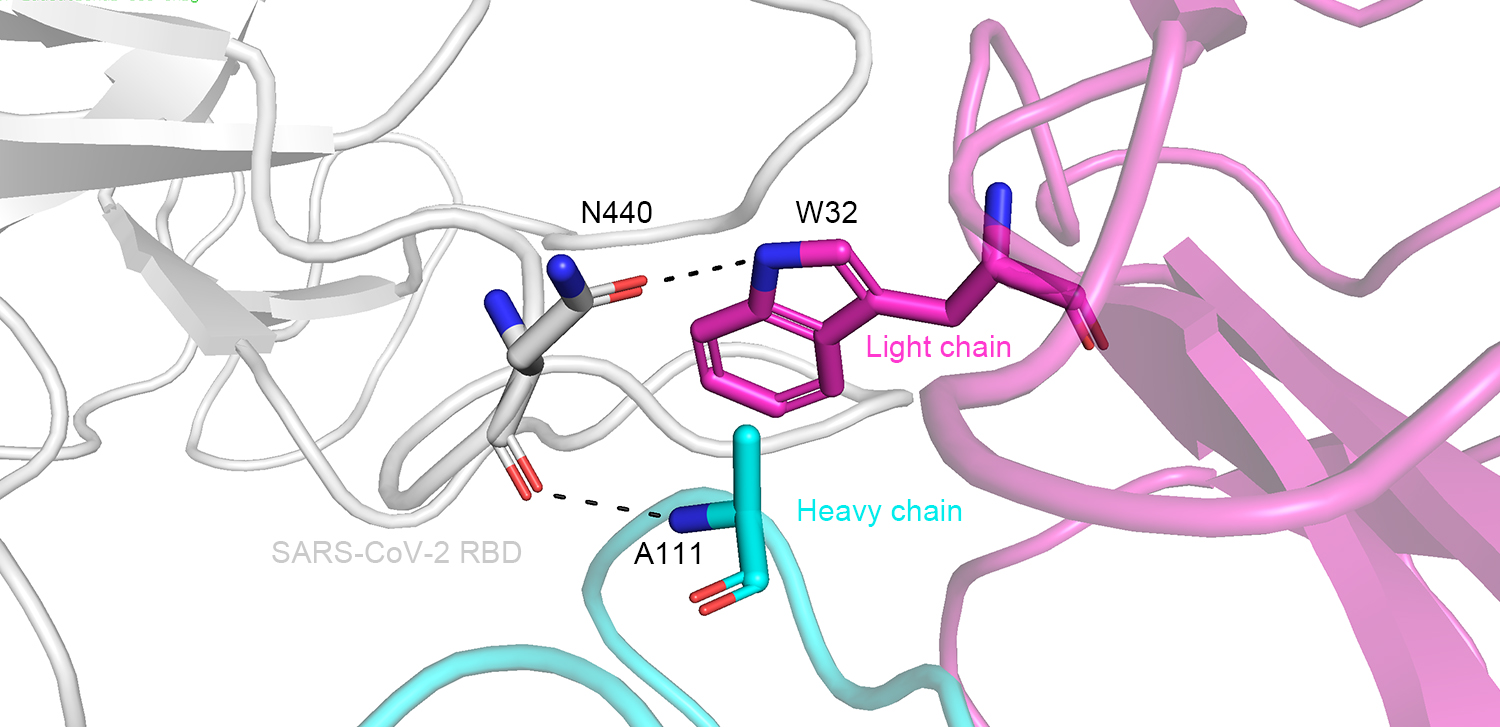

Supplement: Supplemental Material [file TEMI_A_2032374_SM2670.zip › Suppl files/Supplementary Figure 3.jpg]

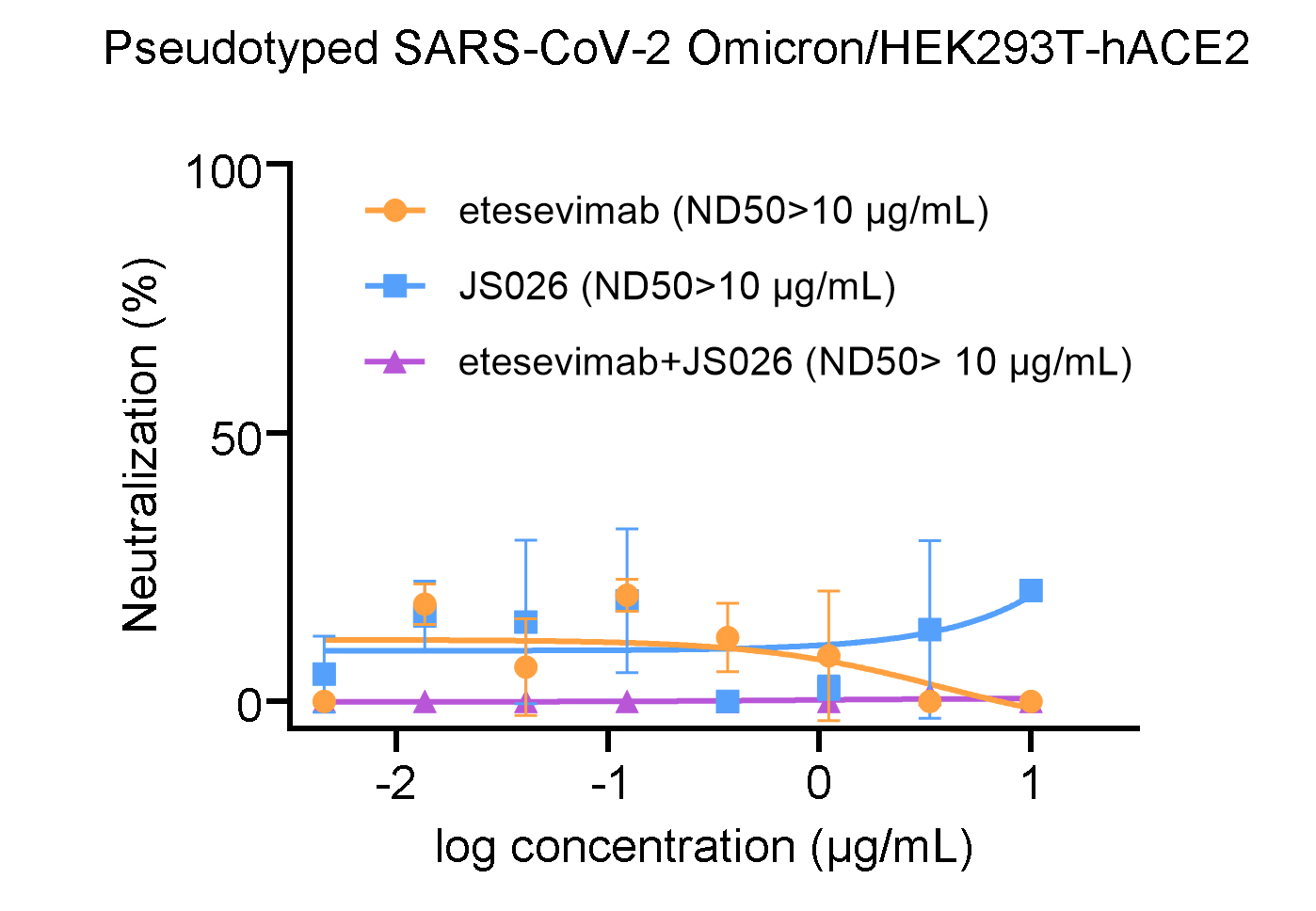

Supplement: Supplemental Material [file TEMI_A_2032374_SM2670.zip › Suppl files/Supplementary Figure 4.jpg]
